# Characteristics and outcome of ill critical patients with influenza A infection

**DOI:** 10.11604/pamj.2018.29.174.13098

**Published:** 2018-03-26

**Authors:** Rania Bouneb, Manel Mellouli, Houda Bensoltane, Jamila Baroudi, Imed Chouchene, Mohamed Boussarsar

**Affiliations:** 1 Department of Intensive Care Unit, University Hospital of Farhat Hached, Susah, Tunisia; 2 Department of Preventive Medicine, Faculty of Medicine, Susah, Tunisia; 3 Department of Emergency, University Hospital of Farhat Hached, Susah, Tunisia

**Keywords:** H1N1, influenza virus, intensive care unit, risk factors, symptoms, prognosis, treatment

## Abstract

**Introduction:**

To describe all patients admitted to Tunisian intensive care unit with a diagnosis of influenza A/H1N1 virus infection after the 2009 influenza pandemic and to analyse their characteristics, predictors of complications and outcome.

**Methods:**

All patients with influenza > 18-years-old hospitalized to the ICU department of Tunisian University hospital of Sousse, between December 1, 2009 and March 31, 2016, with a positive influenza A/H1N1/09 reverse transcriptase polymerase chain reaction (RT-PCR) from a nasopharyngeal specimen were included, were included.

**Results:**

40 cases were admitted to intensive care units. During the reporting period, 22 deaths in intensive care units (55%) were reported, the median age was 53 years (IQR 37-61), 24 (61%) were male, The median scores SAPS II and SOFA were respectively 29 (IQR 23-36) and 6 (IQR 3-10), 27% had chronic obstructive pulmonary disease (COPD), 33.3% diabetic and no patients were vaccinated against influenza A. The cause of admission was in 72.5% of the cases was hypoxemic pneumonae. By using a logistic regression, we found after adjustment to age, that acute respiratory distress syndrome (ARDS) (OR = 27; 95%CI: 3.62-203.78) was the only factor significantly associated with severe outcomes of the cases.

**Conclusion:**

Patients in the first post pandemic season were significantly older and more frequently had underlying medical conditions. Multivariate analysis showed that older male patients with chronic lung disease were at increased risk for a severe clinical outcome.

## Introduction

Influenza pandemics have been associated with increased illness and death. Each pandemic is different, and areas of uncertainty always exist when an influenza virus emerges and becomes pandemic. In April 2009, the novel influenza A (H1N1) pdm09 virus emerged in Mexico and then spread rapidly throughout the world [1]. Influenza is generally a self-limiting infection with systemic and respiratory symptoms that usually resolve after 3-6 days. Most persons infected with the 2009 influenza A (H1N1) pdm09 virus experienced uncomplicated illness with full recovery within 1 week, even without medical treatment; severe progressive disease developed in only a small subset of patients [2]. Primary viral pneumonia was the most common finding in severe cases, but secondary bacterial infections played a role in ≈30% of fatal cases [3]. Hospitalized patients were often affected by other medical conditions, such as diabetes and cardiovascular, neurologic and pulmonary diseases [4]. Advances in therapy for malignancies, autoimmune disorders and end-stage organ diseases have led to improved survival, but also to an increase in the number of immunosuppressed patients. These patients are particularly at risk for opportunistic and community-acquired infections, such as respiratory virus infections, resulting in considerable illness and death [5]. Although patients hospitalized with pandemic influenza A (H1N1) pdm09 infection had substantial severe illness, the overall number of deaths was lower than reported in the earliest studies. The overall number of deaths caused by influenza A (H1N1) pdm09 infection was similar to that caused by seasonal influenza and lower than that of previous pandemics [6]. The most common cause of death was respiratory failure [7]. Other reported causes of death included pneumonia, high fever leading to neurologic sequelae, dehydration from excessive vomiting and diarrhea and electrolyte imbalance. Severe cases were most frequent in middle-aged patients, who often had coexisting conditions [7]. Although to date there seems to be no major difference between the virulence of influenza A (H1N1) pdm09 strains and seasonal influenza [8] strains, a more aggressive course in specific populations, such as in young patients and pregnant women, has been reported [8, 9]. Further risk factors include obesity, chronic lung disease, chronic heart disease, chronic renal disease, diabetes mellitus, and severe immunosuppression [4, 10, 11]. Contradictory findings have been reported in regard to varying disease severity during the pandemic season. Although some researchers did not observe any differences in disease severity between the first and second pandemic outbreaks in 2009 [12, 13], another study showed a 4-fold increase in hospitalization and a 5-fold increase in number of deaths in the second wave [14]. However, disease severity of postpandemic seasons has been rarely analyzed. We performed a retrospective analysis of all patients with laboratory-confirmed influenza A (H1N1) pdm09 virus infection who were hospitalized at the Tunisian University Hospital, in the postpandemic season 2010-16 to identify possible risk factors associated with severe clinical outcome.

## Methods

We conducted a retrospective cohort study of all patients admitted to the University Hospital, with laboratory-confirmed influenza A (H1N1) pdm09 infection from October 2010 through April 2016. We defined a case-patient as a hospitalized person with influenza-like illness and influenza A (H1N1) pdm09 virus infection confirmed by real-time PCR. Microbiologic studies, hospital and ICU admission criteria and treatment decisions were not standardized but made at the discretion of the attending physicians.

**Data collection**: We collected data on demographic characteristics, coexisting conditions, clinical signs and symptoms, biochemical analyses, chest radiograph findings, antiviral and antibacterial therapy, concomitant and secondary bacterial infections as well as outcome, including death. Pneumonia was defined as the presence of a new infiltrate shown on a chest radiograph plus fever (temperature >38°C) and respiratory symptoms. Bacterial infections were diagnosed by means of blood cultures and analysis of sputum or bronchoalveolar lavage specimens. Routine laboratory analyses included C-reactive protein level and leukocyte count.

**PCR**: nasopharyngeal samples and bronchoalveolar lavage specimens were collected from the patients and either processed within 2 hours or refrigerated at -20°C. PCR based detection of influenza A (H1N1) pdm09 and seasonal influenza A and B strains was performed by 1-step real time reverse transcription PCR (16). Total RNA from respiratory samples was isolated with the QIAamp Viral RNA Mini Kit, according to the manufacturer's protocol and reverse transcribed by using random hexamers from the Transcriptor First Strand cDNA Synthesis Kit . Reverse transcription PCR analysis was performed with 5.0 μL RNA by using the RNA Virus Master Kit (Roche,) and the Light Cycler 480 System (Roche) under the following conditions: 8 min at 50°C; 30 s at 95°C; 50 cycles of 5 s at 95°C, 20 s at 60°C and 1 s at 72°C. The primer pairs were used at a concentration of 10 μM.

**Statistical analysis**: We summarized demographic and clinical data for time and severity. Frequencies were compared by using χ2 test or Fisher exact test for categorical variables and the Student t test or Mann-Whitney U-test for continuous variables, as appropriate. Identified risk factors with a p value < 0.2 in the univariate analysis were included in a multivariate logistic regression model to assess independent association with severity. In a stepwise backward procedure, exposures with p > 0.05 were excluded from the model. All comparisons with p < 0.05 were considered statistically significant.

## Results

Descriptive Epidemiology We identified 40 hospitalized patients in the postpandemic season (December 2010-March 2016), included in the study group. Demographic and clinical characteristics of influenza patients are shown in Table 1. In univariate analysis, severity of disease was significantly associated with obesity, presence of acute respiratory distress syndrom (ARDS) and hemodynamic failure (Table 2). All patients had respiratory failure at admission; respiratory involvement was severe in 22 (55%) patients with ARDS. Hemodynamic failure was present in 32% of cases and renal failure in 15%. Initial ventilatory management of ARDS patients was performed in invasive ventilation for 22 (55%) patients were divided into 13 (32.5 %) Severe ARDS, 12 (30%) Moderate ARDS and 15 (37.5%) severe. The median duration of ventilation was 4 (IQR 2-7) days. The median PaO2/FiO2 ratio was 120 mmHg (IQR 67-200). 18 (45%) patients required the use of curare, 2 (5%) were positioned in DV. Only one pregnant patient needed extracorporeal circulatory assistance. Seventeen% patients (42.5%) had been treated with Oseltamivir. Antibiotic therapy was administered in 33 (82.5%) patients in the initial phase. Systemic corticosteroid therapy was administered to 17 patients (42.5%). The median length of stay in intensive care units was 4.5 days (IQR3-7.75). The mortality was observed in 55% (22 patients) in intensive care units. In logistic regression and after adjustment to age, the only factor associated with death was the occurrence of ARDS (Table 3).

**Table 1:** Demographic and clinical characteristics of influenza infected ICU patients

Characteristic	Total admitted patients=40
Age (Med, IQR _25-75_)	53 (37-61)
Male Sex (n, %)	24(60)
**Underlying medical condition(n, %)**	
Immunosuppression	0(0)
Chronic lung disease	12(30)
Cardiovascular disease	12(30)
Renal impairment	2 (5,12)
Diabetes	13 (33,3)
Metabolic dysfunction	2 (5,12)
Pregnant (n, %)	2 (5,12)
Obesity (IMC>30), n (%)	2(5.12)
Vaccination	12(30)
**Reason for hospitalization** **(n, %)**	
Hypoxemic Pneumonia without shock	12(30)
Hypoxemic Pneumonia with Shock	17(42,5)
Myocarditis with Cardiogenic Shock	1(2,5)
Decompensation of COPD	11(27,5)
Mechanical ventilation (n, %)	29(74.3)
Leukocyte count on admission, /nL† (Med, IQR 25-75)	11100(6575- 15500)
CRP level on admission, mg/L	126(3,5-488)
Delayed hospital admission (Med, IQR 25-75)	7 (5-11)
SAPS II (Med, IQR 25-75)	29(23-36)
SOFA (Med, IQR 25-75)	6(3-10)
Days in ICU (Med, IQR 25-75)	4.5 (3-7,75)
Died (n, %)	22(55)

SAPSII, Simplified acute physiology score; SOFA, Sepsis-related Organ Failure Assessment; ICU intensive care unit

**Table 2:** Factors associated with the death of patients hospitalized in intensive care for infection with the influenza A virus

	Total (n=40)	Survivors (n = 18)	died (n=22)	*p*
Characteristics of the population				
Age (Med, IQR _25-75_)	48(17-76)	49(27-79)	53.5 (17-76)	0.54
Male, n (%)	24(61.5)	12(70.5)	12(54.5)	0.20
Pregnancy, n (%)	2(5)	0(0)	2(9)	-
IMC >30 (kg/m^2^)	12(30.7)	2(11)	10	0.02*
**Comorbidities, n (%)**				
Chronic lung disease	10(25)	5(27)	5(23)	0.43
Diabetes	13(32)	7(38)	6(27)	0.15
Cardiovascular	12(30)	5(27)	7(32)	0.60
Obesity	12(30)	2(11)	10(45)	0.02*
**Severity Score**	****		****	****
SAPSII (Med, IQR _25-75_)	29(6-66)	26(6-32)	36.95(28-50)	<10^-3*^
SOFA (Med, IQR _25-75_ **)**	6(3-10)	3(0-6)	9(4-16)	<10^-3*^
**Failure of Organ**				
Hémodynamique, n (%)	13(33.3)	2(11)	11(50)	0.01*
Rénale, n (%)	6(15)	1(5)	5(23)	0.14
SDRA (P/F<200), n(%)	22(56.4)	3(17.6)	19(86.3)	<10^-3*^
**Données biologiques (Med, IQR _25-75_)**			****	
Creatinine (µmol/l)	90.5 (47-620)	90(64-215)	91(47-620)	0.09
Plates (10^3^élts/mm^3^)	180(56-497)	180(56-497)	180(71-462)	0.55
CRP (mg/l) (Med, IQR _25-75_)	126.5 (3.5-488)	39(3-488)	180(42-337)	0.01*
Glycemia (mmol/l) (Med, IQR _25-75_)	12(7.11-16.25)	11(6.20-16)	12(6.20-18.20)	0.51
Uremia (mmol/l) (Med, IQR _25-75_)	8(5.40-18)	10(6.30-16)	14(8.50-22)	0.48
**Traitement**, n(%)	****			
corticosteroids	17(43.5)	5(29.4)	12(54.5)	0.08
oseltamivir	21(52.5)	9(50)	12(54.5)	0.45
catecholamines	24(60)	5(28)	19(86)	0.004*
Duration VM (Med, IQR 25-75)	4(2-7)	7.57 (2-14)	5.55 (1-11)	0.48
Nosocomial infection, n (%)	8(20)	0(0)	8(36)	0.014*

ARDS, acute respiratory distress syndrom; SOFA, Sepsis-related Organ Failure Assessment; SAPSII, Simplified acute physiology score

**Table 3:** Factors independently associated with severe clinical outcome

Characteristic	Relative risk	(95% CI)	p value
Hemodynamic failure	0,002	[0,00-6,16]	0 ,12
ARDS	27	[3,62-203,78]	0,001*
Nosocomial infection	1	-	0,99
SAPSII	1,07	[0,95-1,2]	0,25
SOFA	1,41	[0,94-2,11]	0,93
CRP	1,01	[1,01-1,26]	0 ,42

ARDS, acute respiratory distress syndrome; SOFA, Sepsis-related Organ Failure Assessment; SAPSII, Simplified acute physiology score

## Discussion

In our study, the rate of severely diseased patients with influenza A (H1N1) pdm09 virus infection increased in the first postpandemic season, resulting in an in-hospital ICU mortality rate of 55%. Also, the length of hospital stay in the postpandemic season and the need for mechanical ventilation and ICU admission were significantly higher than in literature. We identified ARDS as the only independent risk factor of mortality. The influenza A (H1N1) infection in 2009 affected Tunisia nearly 700 000 people (6.5% of the population), 179 patients were hospitalized in various Tunisian resuscitation departments [15]. A recent Tunisian study [15], found that 2008-2009 influenza season in Tunisia was characterized by co-circulation of influenza A/H3N2 (56.25%), influenza A (H1N1) (32.5%) and influenza B (11.25%). In the 2010-11 season, circulating strains were mainly influenza A (H1N1) (70%) and influenza B (22%). The highest prevalence of influenza B was reported in Tunisia (76%). In contrast to findings in the 2009-10 pandemic when highest rates of infection in the community were observed in children. The increase in severe courses of infection and requirement for critical care in 2010-16 might reflect the effects of influenza A(H1N1)pdm09 illness in the remaining susceptible adults and risk groups in the population, primarily older patients with coexisting risk factors for seasonal influenza. Younger age, chronic coexisting conditions, morbid obesity, and bacterial coinfection have been reported as independent risk factors for severe disease in the pandemic season [4]. Of our hospitalized patients with severe influenza A (H1N1) pdm09 infection, 70% had chronic disease. Delayed hospital admission and delayed antiviral therapy have been associated also with an unfavorable outcome in the general population [11]. The time between initial symptoms and admission to hospital is fairly homogeneous, with a median of about 4 days (IQR 2-7). In our cohort was 7 days (IQR 5-11). We identified pneumonia in 29 patients (72.5%). These findings suggest that the presence of pneumonia is a risk factor for poor clinical outcome and death. Studies have shown that the presence of pneumonia in patients infected with the H1N1 influenza virus was a poor prognostic factor and was associated with an increase in the mortality rate [16]. There were also 11 exacerbations COPD of and one case with severe myocarditis. Several studies have described myocarditis as a severe complicating form of influenza [17, 18] and COPD is an independent risk factor for poor clinical outcomes in influenza and hospitalization in intensive care [19].

## Conclusion

In conclusion, our cohort found notable epidemiologic changes and an increased severity and mortality of ICU patients' influenza A (H1N1) pdm09 infections in the postpandemic influenza season. The presence of ARDS at the admission is the only independent factor of mortality. These findings reinforce the need to identify and protect groups at highest risk for adverse outcomes.

### What is known about this topic

- Among recent studies, younger age, chronic coexisting conditions, morbid obesity and bacterial coinfection have been reported as independent risk factors for severe disease in the pandemic season.

### What this study adds

- The presence of ARDS at the admission is the only independent factor of mortality.

## Competing interests

The authors declare no competing interest.
